# Persistence of W135 *Neisseria meningitidis* Carriage in Returning Hajj Pilgrims: Risk for Early and Late Transmission to Household Contacts

**DOI:** 10.3201/eid0901.020131

**Published:** 2003-01

**Authors:** Annelies Wilder-Smith, Timothy M.S. Barkham, Sindhu Ravindran, Arul Earnest, Nicholas I. Paton

**Affiliations:** * Tan Tock Seng Hospital, Singapore; 1 Originated the study idea; was responsible for study design, data collection, analysis, and interpretation; and wrote the final manuscript.; 2 Was responsible for the meningococcal cultures, serogrouping, and pulsed-field gel electrophoresis (PFGE), and contributed to the manuscript.; 3 Performed PFGE and made other contributions to the manuscript.; 4 Was responsible for data entry and statistical analysis.; 5 Contributed to the study design and data interpretation, and co-wrote the final manuscript.

**Keywords:** meningococcal disease, W135 *N. meningitides*, Hajj pilgrims, meningococcal carriage, transmission to household contacts, dispatch

## Abstract

After an outbreak of meningococcal disease caused by *Neisseria meningitidis* W135, associated with the Hajj pilgrimage in 2001, 15% of returning vaccinated pilgrims carried a single W135 clone, and 55% were still carriers 6 months later. Transmission to 8% of their unvaccinated household contacts occurred within a few weeks, but no late transmission took place. Public health interventions are needed to protect household contacts.

The annual Islamic pilgrimage to Mecca and Medina in Saudi Arabia (Hajj pilgrimage) attracts approximately 2 million pilgrims from all over the world. Overcrowding during the 1-month-long religious rituals may facilitate rapid dissemination of meningococci (*1*–*3*). An international outbreak of meningococcal disease caused by *Neisseria meningitidis* W135 occurred in association with the Hajj pilgrimage in 2000 and 2001 (*4*,*5*), with a high attack rate not only among the pilgrims but also among household contacts of returning pilgrims (*6*–*10*).

Meningococcal carriers are the primary source of *N. meningitidis* transmission (*11*). Although vaccination can protect pilgrims against invasive meningococcal disease, it does not prevent acquisition of carriage (*12*). Pilgrims returning from the Hajj may have a high meningococcal carriage rate (*13*), and after the W135 outbreak during the Hajj pilgrimage 2001, we documented a high acquisition rate of a single clone of W135 in pilgrims (*14*). Returning pilgrims may therefore spread the organism to household contacts or even to the community at large.

Although researchers have shown that W135 can attain a high carriage rate (*14*,*15*), no data are available on the duration of carriage of W135. Persistence of carriage may represent a threat to the community and has important public health implications. We set out to determine the persistence of W135 meningococcal carriage in pilgrims and to quantify the ongoing risk of transmission to household contacts.

## Methods

Vaccination records kept at a Moslem center in Singapore were reviewed to identify pilgrims on the Hajj pilgrimage in 2001. Returning pilgrims, all of whom had received pre-Hajj quadrivalent meningococcal polysaccharide vaccine, were contacted and invited to participate in the study.

Tonsillopharyngeal swabs specimens were taken from returning Hajj pilgrims 2 weeks after the Hajj pilgrimage 2001, and those found to be carriers of the W135 clone (henceforth referred to as “pilgrim carriers”) had a repeat swab taken 5–6 months later. Antibiotics were not routinely administered to those who were identified as carriers, although some took antibiotics for other indications during the follow-up.

All household contacts of pilgrim carriers were asked to come for a throat swab within the first month and 5–6 months after the Hajj. In addition, all household contacts whose results of the initial throat swab were negative were asked to return for serial throat swabs taken 2 weeks, 1–2 months, and 2–3 months later.

Swab samples were transferred to a plate of selective culture medium (Oxoid GC, Basingstoke, U.K.). Culture plates were immediately put in candle jars and transferred to the laboratory within 2–4 hours of collection. Serogrouping by latex agglutination (Murex, Dartford, U.K.) and pulsed-field gel electrophoresis (PFGE) was performed on all meningococcal isolates. PFGE was performed by using previously described methods (*16*). The Tenover criteria were used to interpret the pattern of bands (*17*).

All subjects gave written informed consent. The study was approved by the Ethics Committee of Tan Tock Seng Hospital.

## Results

Swab specimens were taken from 373 Malay pilgrims at a median time of 26 days (range 3–45) after return from the Hajj. The median age was 47 years (range 3–78 years), and 57% were female. Of the 373, a total of 61 (16%) were found to be meningococcal carriers (95% confidence interval [CI] 13% to 21%). Of these 61, 56 (92%) were of a single W135 clone by PFGE. Fifteen percent (95% CI: 12% to 19%) of the returning pilgrims were carriers of the W135 clone (Table). Forty pilgrim carriers returned for a repeat swab at 5–6 months (range 144–182 days) after the Hajj. Of those 40, 22 (55%) remained carriers of the W135 clone.

**Table:** Meningococcal carriage rates in returning Hajj pilgrims and household contacts of pilgrim carriers




Meningococcal carriage rate	Returning Hajj pilgrims (n=373) (%)	Household contacts of pilgrim carriers^a^ (n=117) (%)

Overall	61 (16.4)	13 (11.1)
		
W135 clone	56 (15.0)	9 (7.7)


^a^A pilgrim carrier is a returning pilgrim with tonsillopharyngeal carriage of the W135 clone.

The household contacts of 42 of the pilgrim carriers (75%) agreed to provide throat swabs specimens. Swabs were taken from 117 (84%) of all existing 139 household contacts at a median time of 32 (range 5–45) days after the Hajj. The median age of household contacts was 14 years (range 1–52 years), and 65.3% were children <18 years of age. Of the 117 contacts, 13 (11%) were meningococcal carriers, and of those carriers 9 (69%) were found to carry the W135 clone (hereafter referred to as “contact carriers”). The 9 contact carriers belonged to eight households, and 11 (19%) of the pilgrim carriers transmitted the W135 clone to at least one household contact.

Of the 104 contacts with initially negative results, 26 had a repeat swab specimen taken at a median interval of 15 days (range 11–27 days) after the initial swab within the first month after the Hajj, and all results continued to be negative. Thirty-one of the 104 persons had repeat swabs taken at 1–2 months (median of 39 days, range 30–60), 32 at 2–3 months (median 65 days, range 62–72), and 79 at 5–6 months (median 160 days, range 144–182) after the Hajj, and none was found to have become a carrier (Figure).

**Figure:**
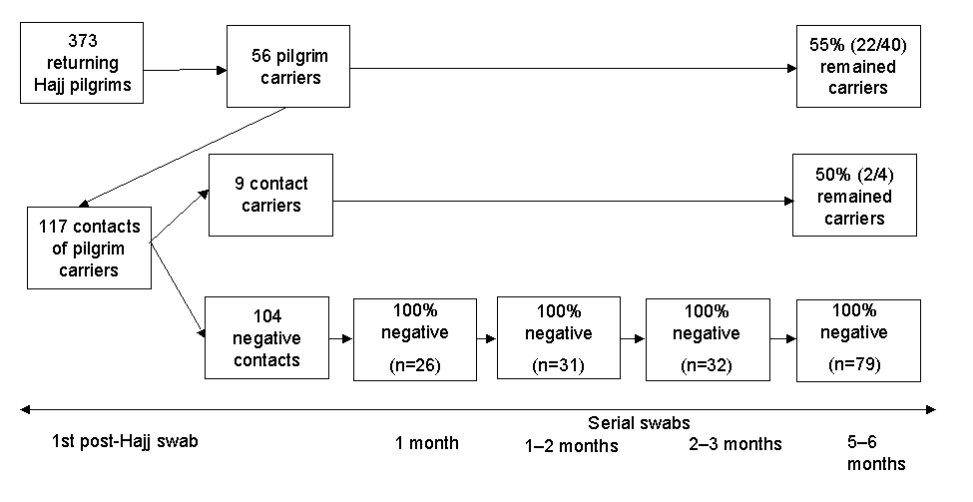
Results of serial tonsillopharyngeal swab specimens from returning Hajj pilgrims and their household contacts. A pilgrim carrier is a returning pilgrim with tonsillopharyngeal carriage of the W135 clone; a contact carrier is a household contact of a pilgrim carrier and carries the W135 clone.

## Discussion

The high meningococcal carriage rate in returning pilgrims is consistent with the rate of serogroup A meningococcal infection found in 14% of pilgrims returning to the United States after the 1987 Hajj (*13*). This rate is also in keeping with studies in other situations in which large numbers of previously unrelated persons have come into close social contact (e.g., university freshmen), and dramatic increases in carriage rate have been shown to occur in the space of just a few days (*18*,*19*). However, the carriage rate of 16% in this study is markedly higher than the 2.6% found in pilgrims returning to the United States after the 2001 Hajj (*20*). This discrepancy is unlikely to be explained by differences in study methodology alone but probably results from differences in living conditions, degree of overcrowding, or social activities during the Hajj. Further detailed epidemiologic studies are warranted to determine exactly which aspect of the pilgrimage is responsible for the high transmission that we have documented. The proportion of overall carriage attributable to this single W135 clone (92% of all carriage) in our study is unusual. Even in the situation of a meningitis outbreak, the proportion of carriage of the hypervirulent strain responsible for invasive disease rarely exceeds the overall proportion of carriage in the population by a few percentage points (*11*,*21*,*22*).

In addition to our findings that W135 can attain a high carriage rate, we have shown that duration of carriage with this strain is lengthy (55% of carriers remain positive after 5–6 months). This longevity indicates that returning pilgrims may represent an ongoing threat to the community.

To our knowledge, this study is the first to investigate the extent of early transmission of *N. meningitidis* from pilgrim carriers to household contacts. We found that returning pilgrims carrying the W135 clone transmitted it to 7.7% of their household contacts, which is of the same order of magnitude as the transmission rate of meningococcal carriage from patients with invasive disease to household contacts (*23*,*24*). However, acquisition occurred only in the first month of contact with the returning pilgrim carriers, and none of the contacts with initially negative results acquired the strain after months of exposure. The absence of late transmission is an important new finding for which the explanation is currently unclear. Possibilities include the absence of particular epidemiologic risk factors for transmission in contacts with persistently negative results, host protective factors, or the attenuated virulence of the organism in the pilgrim carrier over time. Further studies are warranted to investigate our findings.

The absence of late transmission in our cohort is also consistent with our national epidemiologic data, which showed that all the cases of invasive W135 meningococcal disease in contacts of returning Hajj pilgrims occurred within 2 months after the end of the Hajj pilgrimage 2001 and no further cases occurred later in the year (*10*,*25*). However, although persistence of carriage appears not to put the household contact at risk, this persistence may be an important threat to the community at large. Cases of W135 disease were identified in the United Kingdom several months after the end of the Hajj, but most of these case-patients had no identifiable direct contact with Hajj pilgrims (*26*).

Although vaccination may protect the pilgrims from invasive disease, our data show that returning pilgrims represent a sizeable reservoir of a highly transmissible and persistent W135 clone, which places their unvaccinated family contacts (and possibly the community at large) at risk of invasive disease. The appropriate public health response to this problem is unclear. One approach would be to eradicate carriage in pilgrims by administering antibiotics at the point of return to their home countries. However, more data on the impact of this intervention and on resistance and safety issues are needed before embarking on such a large-scale program. Vaccination of household contacts is a potential strategy, but it would be expensive and difficult to implement. Increasing the uptake of the quadrivalent meningococcal vaccine (now mandatory for all pilgrims) may have a beneficial effect in decreasing the carriage of W135 (*6*). Although the vaccine did not prevent acquisition of carriage in our cohort of Singaporean pilgrims, it may have a greater effect when the entire Hajj pilgrim population is vaccinated. Future studies are essental in order to determine the public health impact of such a vaccination program.

## References

[1] al-Gahtani YM, el Bushra HE, al-Qarawi SM, al-Zubaidi AA, Fontaine RE. Epidemiological investigation of an outbreak of meningococcal meningitis in Makkah (Mecca), Saudi Arabia, 1992. Epidemiol Infect. 1995;115:399–409. 10.1017/S0950268800058556 8557071 PMC2271597

[2] Novelli VM, Lewis RG, Dawood ST. Epidemic group A meningococcal disease in Haj pilgrims. Lancet. 1987;2:863. 10.1016/S0140-6736(87)91056-7 2889067

[3] Moore PS, Reeves MW, Schwartz B, Gellin BG, Broome CV. Intercontinental spread of an epidemic group A *Neisseria meningitidis* strain. Lancet. 1989;2:260–3. 10.1016/S0140-6736(89)90439-X 2569063

[4] Taha MK, Achtman M, Alonso JM, Greenwood B, Ramsay M, Fox A, Serogroup W135 meningococcal disease in Hajj pilgrims. Lancet. 2000;356:2159. 10.1016/S0140-6736(00)03502-9 11191548

[5] Meningococcal disease, serogroup W135. Wkly Epidemiol Rec. 2001;76:141–2. 11383502

[6] Hahne SJM, Gray SJ, Aguilera JF, Crowcroft NS, Nichols T, Kacymarski EB, W135 meningococcal disease in England and Wales associated with Hajj 2000 and 2001. Lancet. 2002;359:582–3. 10.1016/S0140-6736(02)07716-4 11867116

[7] Issack MI, Ragavoodoo C. Hajj-related *Neisseria meningitidis* serogroup w135 in Mauritius. Emerg Infect Dis. 2002;8:332–4. 10.3201/eid0803.010372 11927036 PMC2732459

[8] Aguilera JF, Perrocheau A, Meffre C, Hahne S. Outbreak of serogroup w135 meningococcal disease after the Hajj pilgrimage, Europe, 2000. Emerg Infect Dis. 2002;8:761–7. 12141959 10.3201/eid0808.010422 PMC2732506

[9] Fonkoua MC, Taha MK, Nicolas P, Cunin P, Alonso JM, Bercion R, Recent increase in meningitis caused by *Neisseria meningitidis* serogroups A and W135, Yaounde, Cameroon. Emerg Infect Dis. 2002;8:327–9. 10.3201/eid0803.010308 11927034 PMC2732466

[10] Wilder-Smith A, Goh KT, Barkham T, Paton N. Virulence of the Hajj associated W135 outbreak strain: estimates of attack rate in a defined population and the risk of developing invasive disease in carriers. [Q8: update?]. Clin Infect Dis. In press. 10.1086/367858 12627350

[11] Stephens DS. Uncloaking the meningococcus: dynamics of carriage and disease. Lancet. 1999;353:941–2. 10.1016/S0140-6736(98)00279-7 10459897

[12] Rosenstein NE, Perkins BA, Stephens DS, Popovic T, Hughes JM. Meningococcal disease. N Engl J Med. 2001;344:1378–88. 10.1056/NEJM200105033441807 11333996

[13] Moore PS, Harrison LH, Telzak EE, Ajello GW, Broome CV. Group A meningococcal carriage in travelers returning from Saudi Arabia. JAMA. 1988;260:2686–9. 10.1001/jama.260.18.2686 3184335

[14] Wilder-Smith A, Barkham TMS, Earnest A, Paton NI. Acquisition of meningococcal carriage in Hajj pilgrims and transmission to their household contacts: prospective study. BMJ. 2002;325:365–6. 10.1136/bmj.325.7360.365 12183308 PMC117886

[15] MacLennan JM, Urwin R, Obaro S, Griffiths D, Greenwood B, Maiden MC. Carriage of serogroup W-135, ET-37 meningococci in The Gambia: implications for immunisation policy? Lancet. 2000;356:1078. 10.1016/S0140-6736(00)02734-3 11009146

[16] Bevanger L, Bergh K, Gisnas G, Caugant DA, Froholm LO. Identification of nasopharyngeal carriage of an outbreak strain of *Neisseria meningitidis* by pulsed-field gel electrophoresis versus phenotypic methods. J Med Microbiol. 1998;47:993–8. 10.1099/00222615-47-11-993 9822298

[17] Tenover FC, Arbeit RD, Goering RV, Mickelsen PA, Murray BE, Persing DH, Interpreting chromosomal DNA restriction patterns produced by pulsed-field gel electrophoresis: criteria for bacterial strain typing. J Clin Microbiol. 1995;33:2233–9. 7494007 10.1128/jcm.33.9.2233-2239.1995 PMC228385

[18] Ala’Aldeen DA, Neal KR, Ait-Tahar K, Nguyen-Van-Tam JS, English A, Falla TJ, et al. Dynamics of meningococcal long-term carriage among university students and their implications for mass vaccination. J Clin Microbiol. 2000;38:2311–6. 10834994 10.1128/jcm.38.6.2311-2316.2000 PMC86789

[19] Neal KR, Nguyen-Van-Tam JS, Jeffrey N, Slack RC, Madeley RJ, Ait-Tahar K, Changing carriage rate of *Neisseria meningitidis* among university students during the first week of term: cross sectional study. BMJ. 2000;320:846–9. 10.1136/bmj.320.7238.846 10731181 PMC27326

[20] Centers for Disease Control and Prevention. Update: assessment of risk for meningococcal disease associated with the Hajj 2001. MMWR Morb Mortal Wkly Rep. 2001;50:221–2. 11300626

[21] Caugant DA, Kristiansen BE, Froholm LO, Bovre K, Selander RK. Clonal diversity of *Neisseria meningitidis* from a population of asymptomatic carriers. Infect Immun. 1988;56:2060–8. 3135270 10.1128/iai.56.8.2060-2068.1988 PMC259523

[22] Conyn-van Spaendonck MA, Reintjes R, Spanjaard L, van Kregten E, Kraaijeveld AG, Jacobs PH. Meningococcal carriage in relation to an outbreak of invasive disease due to *Neisseria meningitidis* serogroup C in the Netherlands. J Infect. 1999;39:42–8. 10.1016/S0163-4453(99)90101-9 10468128

[23] Farries JS, Dickson W, Greenwood E, Malhotra TR, Abbott JD, Jones DM. Meningococcal infections in Bolton, 1971–74. Lancet. 1975;2:118–20. 10.1016/S0140-6736(75)90016-1 49706

[24] Simmons G, Martin D, Stewart J, Jones N, Calder L, Bremner D. Carriage of *Neisseria meningitidis* among household contacts of patients with meningococcal disease in New Zealand. Eur J Clin Microbiol Infect Dis. 2001;20:237–42. 11399012 10.1007/pl00011260

[25] Meningococcal disease caused by *N. meningitidis* serogroup W135. Epidemiological News Bulletin Singapore. 2001;27:55–6.

[26] Wilder-Smith A, Barkham TMS, Paton NI. Sustained outbreak of W135 meningococcal disease in east London, UK. Lancet. 2002;360:644–5. 10.1016/S0140-6736(02)09796-9 12241961

